# Disclosing the Interaction of Gold Nanoparticles with Aβ(1–40) Monomers through Replica Exchange Molecular Dynamics Simulations

**DOI:** 10.3390/ijms22010026

**Published:** 2020-12-22

**Authors:** Francesco Tavanti, Alfonso Pedone, Maria Cristina Menziani

**Affiliations:** 1 CNR-NANO Research Center, Via Campi 213/a, 41125 Modena, Italy; 2 Department of Chemical and Geological Sciences, University of Modena and Reggio Emilia, Via Campi 103, 41125 Modena, Italy; alfonso.pedone@unimore.it (A.P.); mariacristina.menziani@unimore.it (M.C.M.)

**Keywords:** gold nanoparticle, computational simulation, amyloid, Alzheimer’s, molecular dynamics

## Abstract

Amyloid-β aggregation is one of the principal causes of amyloidogenic diseases that lead to the loss of neuronal cells and to cognitive impairments. The use of gold nanoparticles treating amyloidogenic diseases is a promising approach, because the chemistry of the gold surface can be tuned in order to have a specific binding, obtaining effective tools to control the aggregation. In this paper, we show, by means of Replica Exchange Solute Tempering Molecular Simulations, how electrostatic interactions drive the absorption of Amyloid-β monomers onto citrates-capped gold nanoparticles. Importantly, upon binding, amyloid monomers show a reduced propensity in forming β-sheets secondary structures that are characteristics of mature amyloid fibrils.

## 1. Introduction

The aggregation of unfolded amyloid-β monomers into rich β-sheet mature amyloid fibrils is one of the key events in the appearance of amyloid plaques, a characteristic hallmark of the Alzheimer’s disease (AD) [1,2,3]. Amyloid-β (Aβ) fibrils are commonly present in the brain under different alloforms, with lengths from 36 to 43 amino acids [4,5]. The most predominant forms are the Aβ(1–40) that comprise the 90% of Aβ secreted by cells, followed by the Aβ(1–42) that are approximately 10%; the latter ones are the most toxic and with higher amyloidogenicity [6]. Aβ are commonly found in unfolded monomeric units and their aggregation into β-sheet structures forming mature fibrils that are arranged into two or three antiparallel β-strands aligned along a principal axis [4,5]. The subsequent aggregation of elongated mature fibrils results in the formation of amyloid plaques that deposit and compromise synaptic connections, resulting in neuronal cell loss [7]. The inhibition of the amyloid folding into β-sheet structures is one of the promising approaches to treat AD [8], and the development of effective inhibitors is of paramount importance. Epidemiological studies have shown that the high intake of flavonoids and polyphenols commonly found in fruit and vegetables [9] can reduce the risk of AD and cognitive impairments [10,11]; however, few of them are currently under investigations in clinical trials [8]. An additional promising approach to control and inhibit amyloid folding is based on the use of nanoparticles (see References [12,13] for more details), which can be employed both as therapeutic and as diagnostic tools for amyloid proteins, such as β_2_-microglobulin [14]. In fact, AuNP with different coatings and sizes have been used to label the polymorphs of several amyloidogenic proteins, such as Aβ(1–40) and α-synuclein, involved in Alzheimer’s and Huntington’s diseases [15]. Moreover, a recent study on the interaction of monolayer-protected gold nanoparticles with Aβ(1–40) fibrils showed that hydrophobic contacts are mainly responsible for the strong absorption of the β-sheets structures [16], and the interactions with gold interfaces can significantly modify the secondary structure of Aβ(1–42) fibrils [17]. 

Understanding the details of the interactions between AuNP and proteins, such as amyloid fibrils, is of critical importance for the rational design of new tools in nanomedicine and nanobiotechnology. In this context, computer simulations can be considered a complementary approach to experimental studies that provide an in-depth view of the system and of the interaction mechanism [16,18,19]. One of the main drawbacks of classical computer simulations is that the reachable time scales are not long enough to describe the folding of the proteins. To overcome this limitation and to reduce the computational effort, enhanced sampling techniques such as Metadynamics or Replica Exchange have been developed [20,21]. In particular, the use of replica exchange molecular dynamics has been found to efficiently accelerate the conformational sampling of amyloidogenic proteins and intrinsically disordered proteins [22,23].

Therefore, in this paper, the interaction of citrate-capped gold nanoparticles (AuNPs) with unfolded Aβ(1–40) fibrils will be studied by the use of extensive replica exchange solute tempering (REST) molecular dynamics simulations. We aim to describe at the molecular level the interactions that modulate the folding of amyloid-β monomers in the presence of AuNPs and the mechanism that hinders the formations of the β-structures characteristic of the mature fibrils typical of AD.

## 2. Results

### 2.1. Binding Contacts

**Figure 1:**
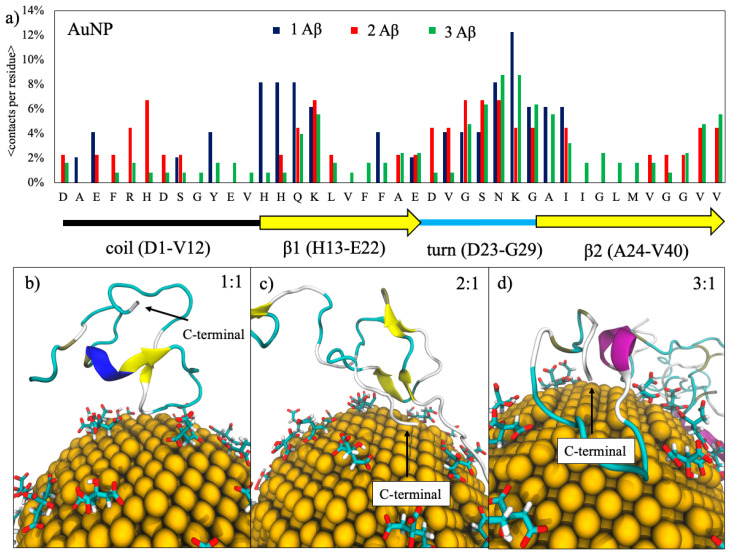
In panel (**a**), the contact probability of Aβ(1–40) peptide residues with the gold nanoparticles (AuNP) for each amyloid-β concentration studied. The dashed line represents the threshold that defines persistent contacts (contact is considered as persistent when a residue has a contact probability greater than 3%). The graphical representation of the secondary structure of the folded mature amyloid-β(1–40) is shown at the bottom of the figure. In panels (**b**–**d**), some representative snapshots of the adsorption of amyloid monomers onto citrate-capped AuNP at the three different ratios, respectively. Proteins are represented in cartoons colored accordingly to their secondary structures highlighting the C-terminal region.

The analysis of the contacts between the Aβ(1–40) and the AuNP observed during the dynamic simulations runs for the three concentration ratios studied (1:1, 2:1, and 3:1 Aβ(1–40)/AuNP) is reported in Figure 1. An interesting trend is shown in Figure 1a, in particular, when considering persistent contacts (contacts are defined as persistent when their probability is greater than 3%). We observed two well-defined binding sites located at the sequence stretches ^13^HHQK^16^ and ^24^VGSNKGAI^31^. The first one corresponds to the beginning of the β1 β-sheet in the structure of the mature amyloid-β(1–40) fibril, while the second one to the turn region connecting the β1 and β2 β-sheets and is the predominant one for all monomer concentrations. The binding sites located at the elbow and at the β1 β-sheet were previously observed for monolayer-capped AuNPs interacting with Aβ(1–42) and with Aβ(1–40) protofibrils by means of molecular dynamics simulations [16].

When increasing the number of Aβ(1–40) monomers in the simulation box, the binding sites become more spread, with the appearance of an additional site located at the C-terminal region of the fibril corresponding to the stretch of amino acids ^36^VGGVV^40^, while the probability of interactions with amino acids from 1 to 14 and from 17 to 24 decreases. The difference in the contact probability at a higher protein concentration is given by the monomer–monomer interaction. Amino acids 1–14 and 17–24 are buried in the inner region of the amyloid aggregates formed, with a consequent decrease in the contact probability. Moreover, the appearance of a new contact site in the C-terminal was found only for the 2:1 and 3:1 ratios where the ^36^VGGVV^40^ amino acids lie on the AuNP surface, while, in the 1:1 ratio, the C-terminal remains far from the AuNP, as shown in Figure 1.

**Figure 2:**
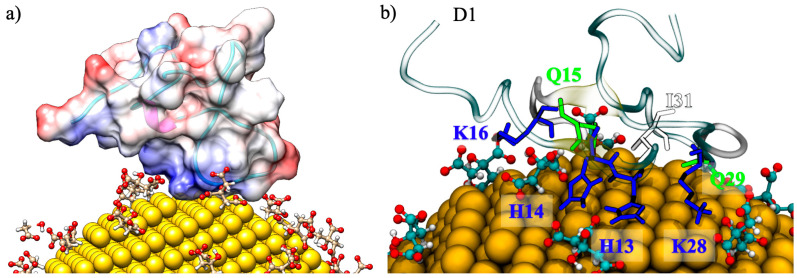
Representative snapshots of Aβ-peptides interacting with the citrate-capped AuNP; the results for the 1:1 Aβ(1–40)/AuNP concentration ratio are shown. In panel (**a**), the Aβ is rendered as a surface colored accordingly to its coulombic potential; negatively charged residues are colored in red, and positively charged residues are shown in blue. In panel (**b**), the interaction driven by positively charged amino acids is shown in blue.

These preferential binding sites of Aβ(1–40) with AuNP are observed since the first step of the interaction between the positively charged surface of the amyloid-β peptides and the negatively charged citrates on AuNP. In this step, called the recognition step, the positive region of the fibril is found to interact directly with citrates at the surface of the AuNP, as shown in Figure 2a, where the electrostatic potential mapped on the amyloid surface is reported. A similar behavior was previously observed in the recognition step of the interaction between monolayer-capped AuNPs, where positively charged amino acids R5 and H6 grab the negatively charged tails of the capping-ligands, driving the adsorption of AuNP [16]. The role of the electrostatic interactions is identified as being one of the driving forces for protein–nanoparticle interactions, although the repulsive or attractive nature of the interaction depends both on the covering of the NP and on the primary, secondary, and tertiary structures of the protein [24,25,26,27,28]. A detailed view of a representative binding mode is given in Figure 2b, where the interactions between the K28, H13, H14, and K16 amino acid residues of the Aβ(1–40) and the adsorbed citrates obtained for the MD simulations of the 1:1 Aβ(1–40)/AuNP ratio are highlighted. After a few ns of MD simulation, when the electrostatic recognition is accomplished, additional amino acids (G26, Q15, Q29, and I31) establish Van der Waals interactions directly with the gold atoms stabilizing the binding pose of amyloid for the remaining simulation time. In fact, the Aβ-peptides never lie down over the AuNP surface but remain pointed outward, with only a few residues in contact with the AuNP.

It is worth highlighting that the physicochemical characteristics of the functionalized surface of AuNP are mainly responsible in driving the interactions with the proteins [24,26]. In fact, recent works on the interactions of monolayer-protected AuNP with preformed amyloid Aβ(1–40) and Aβ(1–42) fibrils [15,16], where the NP coating was made by neutrally charged, hydrophobic and hydrophilic ligands showed that, in this case, the hydrophobic interactions acted as diving forces for the stable binding of Aβ fibrils onto the AuNP.

### 2.2. Structural Analysis

**Figure 3:**
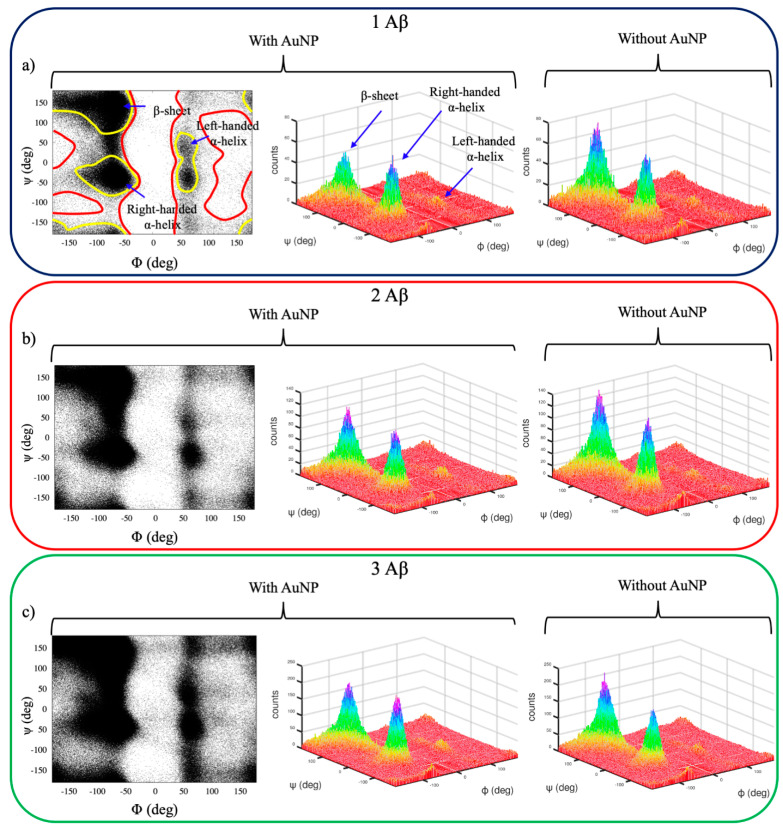
2D and 3D Ramachandran plots obtained by sampling the dihedrals of the eight replicas of each Aβ(1–40) peptide during the 100-ns replica exchange solute tempering (REST) simulations with and without the presence of AuNP. The results for Aβ in the 1:1, 2:1, and 3:1 Aβ(1–40)/AuNP concentration ratios are reported in panels (**a**–**c**), respectively. In the 2D plots, the regions delimited by yellow lines are the most populated and are associated with a well-defined secondary structure, while the regions delimited by red lines are poorly sampled. In the 3D plots, the most probable structures are colored in purple, while the least probable structures are colored in red. Φ and Ψ are defined as the N_i−1_, C_i_, Cα_i_, and N_i_ and the C_i_, Cα_i_, N_i_, and C_i+1_ torsion angles, respectively.

Figure 3 shows the Ramachandran plots [29] of the Aβ(1–40) monomers for the concentration ratios 1:1, 2:1, and 3:1 Aβ(1–40)/AuNP and for the monomers alone, obtained by sampling the dihedrals of eight replicas of each peptide during the 100-ns REST simulations, for a total of 8000 configurations sampled for each monomer ratio. The Φ and Ψ are defined as the N_i−1_, C_i_, Cα_i_, and N_i_ and the C_i_, Cα_i_, N_i_, and C_i+1_ torsion angles, respectively.

Unfolded Aβ monomers sample all the allowed regions of the Ramachandran plot, which are delimited by yellow lines in Figure 3a. Monomers fold preferentially in both right-handed α-helices and β-sheet secondary structures, while left-handed α-helices are uncommon (Figure 3a, 3D plot). It is worth noting that the distribution of β-sheets of monomers alone in a solution (Figure 3a, right panel) is more pronounced with respect to the one for α-helices; the opposite holds for the interacting monomers, suggesting a moderate ability of AuNP to inhibit β-sheets formation. This is observed for all the concentration ratios, although broader distributions for 2:1 and 3:1 (Figure 3b,c) are found.

These results demonstrate that REST simulations are able to span all possible amyloid conformations, avoiding forbidden regions that are related to unphysical protein conformations. The analysis of the Ramachandran plots also suggests that the distribution of the secondary structures spanned mainly swings between α-helices and β-sheets.

**Figure 4:**
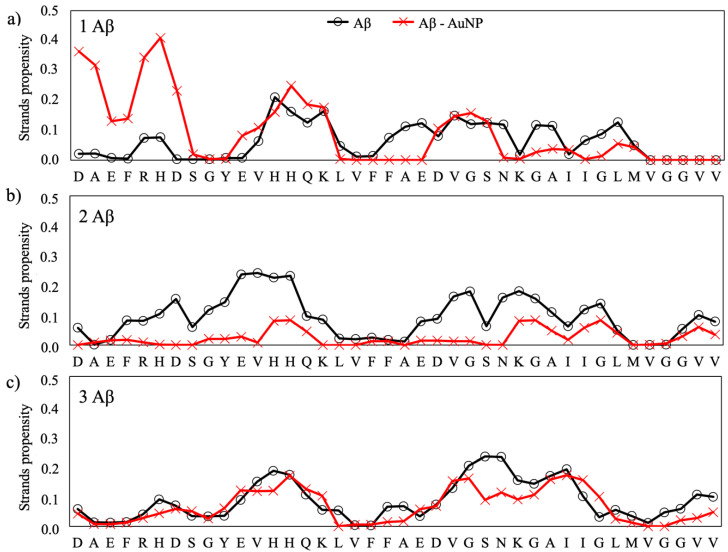
β-strand propensity for each residue of the Aβ(1–40) peptides for the three ratios: 1:1 (**a**), 2:1 (**b**) and 3:1 (**c**).

**Figure 5:**
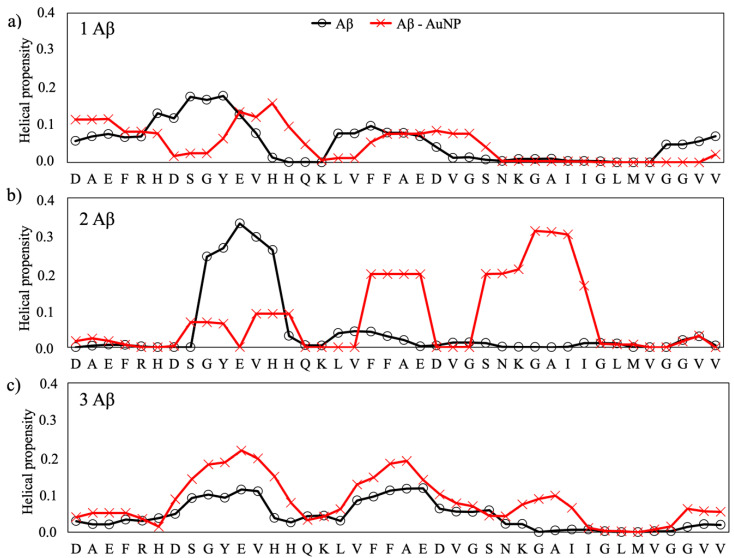
Helical propensity for each residue of the Aβ(1–40) peptides for the three ratios: 1:1 (**a**), 2:1 (**b**) and 3:1 (**c**).

The secondary structure propensities of the Aβ peptides, expressed as the β-strand and a-helix, for each amino acid of the isolated and interacting peptides are reported in Figure 4 and Figure 5, respectively. The secondary structure data values for the reference structure, the isolate peptides, is taken from our previous work [19], where we studied, by using the same computational conditions as reported in the present work, the early steps of the folding behavior of Aβ monomers towards mature fibrils and the influence on the aggregation process of the monomer concentration and of some natural flavonoids [19]. In that paper [19], we found that the single monomer folds into a compact structure stabilized by hydrophobic contacts, with antiparallel β-strands formed, in particular, in the region ^10^TEVHHQKLVFFAEDVGSNKGAI^31^ (Figure 4), while it shows helical propensity in the regions ^5^RHDSGYEV^12^ and ^16^KLVFFAED^22^, as shown in Figure 5. The same propensities for β-strands and α-helix folding are observed when two and three monomers are present contemporaneously in the simulation box, with the difference that both intra- and intermonomer β-strands are formed. This phenomena was assumed to represent the first steps of the formation of the U-shaped β-stranded amyloid fibrils that lead to the pathological amyloidogenic process [19] and is consistent with the results reported by Cheon et al. [30] for the Aβ 17–42 monomers.

The overall effects of the citrate-capped AuNP is to decrease the strand propensity and increase the helical propensity of the amyloid peptides. In fact, by comparing the propensity of each amino acid to form secondary structures with respect to the isolated Aβ, we observed that AuNP has a net tendency of lowering the propensity for β-strands in the 2:1 and 3:1 Aβ(1–40)/AuNP concentration ratios.

The effect of AuNP on the helical content of Aβ(1–40)/AuNP is modest for the 1:1 ratio (Figure 5a), whereas it is significant for 2:1 and 3:1 Aβ(1–40)/AuNP, with an increase of the helical content in the N-terminal region when two Aβ monomers are present in the simulation box (Figure 5b) and in the ^8^SGYEVH^13^ and ^23^DVGSNKGAI^31^ regions for the 3:1 ratio (Figure 5c). 

### 2.3. Free-Energy Calculations

**Figure 6:**
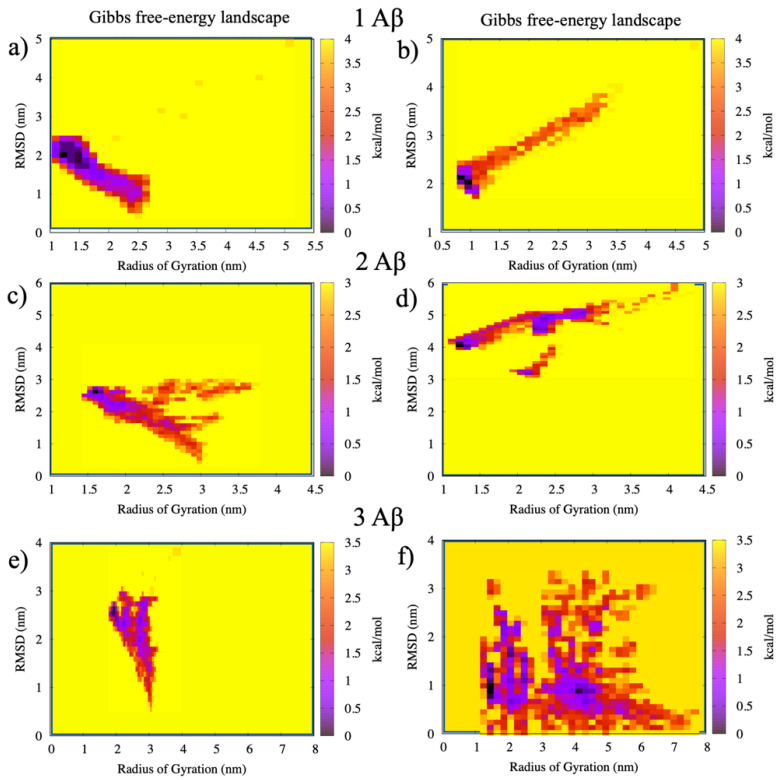
Free-energy surfaces (FES) of the Aβ monomers: in panels (**a**,**b**), the single monomer, in panels (**c**,**d**), the two monomers, and in panels (**e**,**f**), the three monomers without (left panels) and with (right panels) AuNP, respectively. RMSD: root mean square displacement.

The calculation of the free-energy surface (FES) for the Aβ(1–40)/AuNP interactions in the 1:1, 2:1, and 3:1 concentration ratios were obtained using the Radius of Gyration (R_g_) of the Aβ and its root mean square displacement (RMSD) as reaction coordinates. The results are reported in Figure 6, where the right bars represent the color energy scale in kcal/mol in each panel. The minima in Figure 6 represent the most stable configurations of the monomers with and without the presence of AuNP. The tails in the free energy are related to the movements of the monomers before and after the binding, and they are characterized by a broad distribution of the R_g_ values. This trend is consistent with the structural rearrangements of the monomers that pass from the unfolded and extended configurations at the beginning of the simulation to the more compact structures.

It can be observed that the average value of R_g_ increases with the number of Aβ monomers due to the increased size of the system, going from a minimum of 1.3 nm to a maximum of 2.3 nm for one and three monomers noninteracting with AuNP. Thus, the average value of R_g_ for the Aβ(1–40) fibril alone, 1.3 nm, is in agreement with previous computational [17] and experimental [31] results for the Aβ(1–42) fibrils. 

After the interactions with AuNP, the FES appear more complex. In particular, the cases of 2:1 and 3:1 Aβ(1–40)/AuNP show lower energy configurations corresponding to the violet minima in Figure 6 in regions far from the ones of the corresponding monomers, suggesting that the presence of AuNP greatly alters the amyloid aggregation behavior and the formation of mature amyloid fibrils. In particular, the binding with AuNP shifts the free-energy minima to higher values of R_g_, suggesting a more extended structure with respect to the free monomers. This trend can be observed in particular for the 3:1 case, where an additional minimum appears at R_g_ = 4.2, with the three monomers in an extended configuration. 

## 3. Discussion

The aggregation of unfolded amyloid-β monomers into mature amyloid fibrils is a crucial step to understand and to treat Alzheimer’s disease. In silico aggregation studies suffer from the important limitation concerning the very short time scale for the equilibration of just encountered amyloid peptides to stable fibers. This limitation is enhanced by the significantly higher of in silico protein concentration (in the order of 1 mM) than the concentration of amyloidogenic peptides of in vitro (in the μM range) and in vivo studies (in the pM to nM range). For this reason, the use of enhanced sampling techniques is of fundamental importance to accurately describe the free-energy surfaces of amyloid proteins [32]. Here, we employed Replica Exchange Solute Tempering to simulate the interaction of unfolded monomers with citrate-capped gold nanoparticles. The use of AuNP is not only because they can be functionalized with ligands, molecules, and proteins but, also, because of their nanometric sizes allowing them to easily penetrate the blood–brain barrier, enhancing the cellular uptake [33], which is of fundamental importance to deliver drugs to the brain—in particular, for neurodegenerative diseases. Interestingly, we observed that the interactions with the NP are driven by electrostatic forces established between charged amino acids and citrates. As a consequence of these interactions, the peptide strand propensity, characteristic hallmarks of the formation of mature folded fibrils, decreases and the helical propensity increases, and this trend is particularly clear for the 2:1 and 3:1 ratios. For the 1:1 ratio, the AuNP increases the β-strand probability in the N-terminal region due to the high mobility of this tail, but the probability is very small in the sequence ^16^KLVFFAE^22^, which is considered the main one responsible for β-strand aggregation. Moreover, the free-energy surface analysis shows that AuNP perturbs the amyloid behavior with detrimental consequences on the primary steps of mature amyloid fibril formation.

All these findings clearly show that citrate-capped AuNP are able to interfere with the normal aggregation process of unfolded amyloid shifting the secondary structures from β-sheets to helix structures, opening the way to new approaches to treat Alzheimer’s disease.

## 4. Materials and Methods

The GROMOS 54a7 force field [34] was used to perform all Molecular Dynamics simulations. The protonation state of the citrates was set to pH 7, and the citrate force field parameters in the Gromacs format [35,36] were assigned using the Automated Topology Builder [37,38] web server.

**Figure 7:**
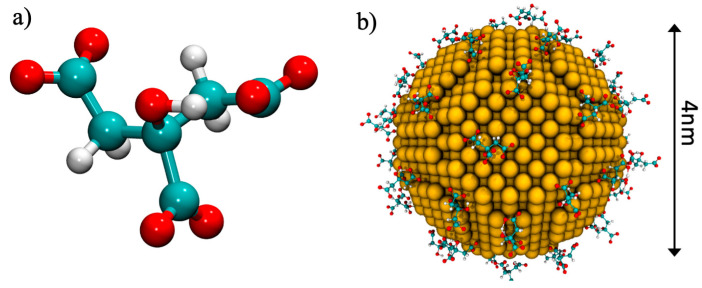
(**a**) The citrate molecule represented in ball-and-stick, and (**b**) the 4-nm diameter AuNP covered by citrates.

The structural model of the amyloid Aβ(1–40) monomers in the unfolded state were retrieved from the Protein Data Bank [39] (PDB ID: 2M9R [40]). For the whole protein, standard protonation states were assigned to ionizable amino acids corresponding to pH 7. The single monomer has a total charge of −3e, which is given by: 3 Glu (−1e), 3 Asp (−1e), 1 Arg (+1e), and 2 Lys (+2e), while the histidine amino acids were assigned a neutral charge. The gold nanoparticles were built by means of the Inorganic Builder plugin of VMD, starting from a truncated octahedron, which was predicted to be one of the thermodynamic equilibrium shapes [41]. The gold core was approximated by a truncated octahedron with a face-centered cubic (*fcc*) crystalline structure containing a fixed number of 2779 gold atoms with a neutral charge. The average diameter of such a nanoparticle core is 4.0 nm, as shown in Figure 7. The AuNP was covered by a number of citrates in order to reproduce the experimental conditions, as previously described [24,42]. To cap the AuNP with citrates, a single AuNP was placed in a simulation box, and 100 citrate molecules were added. A NVT simulation without solvent was performed until a stable number of citrates adsorbed over the AuNP; obtaining 57 citrates capped the AuNP, as shown in Figure 7.

Three simulation boxes were built containing one, two, and three monomers, respectively, as shown in Table 1. The side of the simulation box (9.0 × 9.0 × 9.0 nm) was chosen in order to include the full-length monomer in its unfolded state. The AuNP was positioned at the center of the simulation box and one to three monomers were added in random positions.

Each box contained about 20,000 simple point charge water molecules [43]. Counter ions (Na^+^ and Cl^−^) were added at random locations to neutralize the systems, with an ion concentration of 150 mM, close to the physiological value, obtaining a neutral-charged system.

Each system was first minimized, until the maximum force applied to each atom was smaller than 1 kJ/(mol·nm). Then, the system was equilibrated for 2 ns in the NVT ensemble, where the temperature was controlled using a velocity-rescaling thermostat with a coupling time of 0.1 ps. Then, a NPT equilibration of 2 ns was performed using the Berendsen barostat. Lastly, the production run was performed using the Parrinello–Rhaman barostat with a coupling time of 2 ps and an isothermal compressibility of 4.5 × 10^−5^ bar^−1^ with a timestep of 2.0 fs. The particle-mesh Ewald algorithm was used to calculate the long-range electrostatics [44], with a fourth-order cubic interpolation, a grid spacing of 0.16 nm, and a real space cut-off of 1 nm [45]. Both Van der Waals and neighbor list cut-offs describing short-range interactions were set to 1.0 nm. Data analysis was performed using the GROMACS package [36].

### 4.1. REST Simulations

Replica Exchange Solute Tempering was used in order to enhance the sampling of the protein configurations, as previously done for the Aβ fibrils [46]. In the REST simulations, several replicas of the system are built by assigning different temperatures to the solute while keeping the solvent at a constant temperature. REST simulations follow the same rules of standard Replica Exchange Molecular Dynamics (REMD) simulations, where each given timestep of the replicas are exchanged in temperatures if they satisfy the detailed balance condition, which preserves the Boltzmann distribution at each temperature. With respect to REMD, REST has the advantage to employ less replicas (four to five times less) without compromising the temperature range or the efficiency of the random walk. For this reason, we built 8 replicas with temperatures ranging from 300 K to 440 K with steps of 20 K, and replica exchanges were attempted each 2 ps. The REST simulations are 100-ns-long, for a total of 800 ns of simulations. A sample of the REST trajectory can be found in the Supplementary Materials.

### 4.2. Analysis

The secondary structure contents for Aβ were computed using the DSSP algorithm [47].

The PDB2PQR was used to prepare the monomer structures for the calculations of the electrostatic surface [48], and then, the APBS package (Adaptive Poisson-Boltzmann Solver) [49] was used to compute the electrostatic surface of the amyloid monomers.

The calculation of the free-energy surface (FES) of Aβ monomers with AuNP was obtained using the Gromacs tool “sham”, choosing as reaction coordinates the Radius of Gyration (Rg) of the Aβ monomers and their root mean square displacement (RMSD) during the simulation time. Both the Rg and the RMSD were computed over the whole trajectory of the REST simulations in order to obtain a complete view of the folding process.

## Figures and Tables

**Table 1:** Composition of each simulation box. AuNP: gold nanoparticles.

System	Ratio 1:1	Ratio 2:1	Ratio 3:1
Monomers only [19]	1 monomer	2 monomers	3 monomers
AuNP	1 monomer + 1 AuNP	2 monomers + 1 AuNP	3 monomers + 1 AuNP

## Supplementary Materials

The supplementary materials containing the input files for the REST molecular dynamics and two samples of the trajectory in the Gromacs format can be found at https://www.mdpi.com/1422-0067/22/1/26/s1.
